# A Randomized Control Trial Comparing the Efficacy of Ultrasound-Guided Erector Spinae Plane Block With Thoracolumbar Interfascial Plane Block for Lumbar Spine Surgeries

**DOI:** 10.7759/cureus.70478

**Published:** 2024-09-30

**Authors:** Manisha Ratnam, Krishnamoorthy Karthik, Vishak M Bhaskar

**Affiliations:** 1 Anesthesiology, SRM Medical College Hospital and Research Centre, SRM Institute of Science and Technology, Kattankulathur, IND

**Keywords:** erector spinae plane block (espb), lumbar spine surgery, opioid, thoracolumbar interfascial plane block, ultrasound-guided, visual analog scale (vas) for pain

## Abstract

Background

Lumbar spine surgeries often involve significant postoperative pain, necessitating effective analgesic strategies. Erector spinae plane block (ESPB) and Thoracolumbar interfascial plane block (TLIPB) have emerged as promising regional anesthesia techniques for postoperative pain management. This study compares the efficacy of ultrasound-guided ESPB and TLIPB in providing analgesia following lumbar spine surgeries.

Materials and methods

A randomized controlled trial was conducted with 60 participants undergoing lumbar spine surgeries. Participants were randomized into ESPB and TLIPB, with 30 patients in each group using computer-generated random numbers with odd numbers allotted to Group A and even numbers allotted to Group B. Primary outcomes included postoperative visual analog pain scores, opioid consumption, and duration of analgesia. Secondary outcomes included time to block effectiveness and incidence of adverse events.

Results

Preliminary findings revealed that the ESPB group demonstrated significantly higher opioid consumption during the postoperative period, having Mean±SD 8.30±2.89 mg as compared to 6.43±2.85 mg found in the TLIPB group. Patient visual analog scores were higher in the ESPB group compared to the TLIPB group. Time to first analgesic request was more in the ESPB group, having Mean±SD 505.00±167.88 minutes compared to 435.33±181.01 minutes in the TLIPB group, indicating a potentially longer duration of block effectiveness. There were minimal adverse effects, which were similar in both groups.

Conclusion

Ultrasound-guided ESPB and TLIPB are both effective techniques for postoperative analgesia following lumbar spine surgeries. However, TLIP may offer advantages in terms of reduced opioid consumption after the patient starts complaining of pain, while ESPB provids more duration of analgesia as both the dorsal and ventral rami of spinal nerves are blocked. Further research with larger sample sizes is required to validate these findings and elucidate the optimal regional anesthesia technique for lumbar spine surgeries.

## Introduction

Lumbar spine surgeries are critical interventions of global significance. They are performed to address a wide range of conditions, including degenerative disc disease and spinal fractures. Despite numerous advancements in surgical techniques and anesthesia, effective postoperative pain management remains a significant challenge, often necessitating a multimodal approach to achieve desired outcomes [1]. Regional anesthesia techniques, such as erector spinae plane block (ESPB) and thoracolumbar interfascial plane block (TLIPB), have emerged as promising interventions in perioperative pain management for lumbar spine surgeries [2].

The ESPB, initially described by Mauricio Forero et al. in 2016, involves the deposition of local anesthetic in the fascial plane between the erector spinae muscle and the underlying transverse process [3]. This technique provides analgesia over multiple dermatomes, potentially covering the entire lumbar spine region, with a relatively simple and safe approach under ultrasound guidance [4]. On the other hand, in TLIPB, the drug is deposited between the longissimus and multifidus muscles, aiming to provide similar analgesic effects focused on the dorsal rami compared to ESPB, which targets both dorsal and ventral rami [5].

While both techniques have demonstrated promising results in reducing postoperative pain and opioid consumption in various surgical procedures, including thoracic and abdominal surgeries, their comparative effectiveness, specifically for lumbar spine surgeries, remains understudied [2]. Addressing this gap in the literature, this randomized control trial aims to compare and analyze the efficacy of ultrasound-guided ESPB with TLIPB in providing perioperative analgesia for lumbar spine surgeries. By systematically evaluating pain scores, opioid consumption, perioperative complications, and patient satisfaction, this study endeavors to elucidate the optimal regional anesthesia technique for enhancing postoperative pain management and improving surgical outcomes in this patient population.

## Materials and methods

This randomized, controlled, single-blinded clinical trial was done in patients undergoing elective spine surgery under general anesthesia. The sample size for this study was calculated using the formula:

η= (Zα/2 + Z1-β)²(σ1²+σ2²)/ (μ1-μ2)² =(2.33 + 2.58)² (25.0²+19²)/(163-126)² = 23770.5866/1369 =17.13 n1~30, n2 ~30

The formula compares the means of two populations considering significance level (Zα/2), power (Z1-β), and standard deviations (σ1, σ2). Afterward, 60 patients were randomly allocated into two groups with the help of computer-generated numbers with 30 patients in each group, odd numbers were allotted to Group A, and even numbers were allotted to Group B. The study period was three months, from March 2024 to May 2024. Patients of age group 18-65 years of either sex having American Society of Anesthesiologists (ASA) Grade 1 and 2 undergoing elective lumbar spinal surgery were included in the study. Patients having ASA Grade 3 and 4, deranged coagulation profiles, and who were allergic to local anesthetic agents were meticulously excluded from the study, ensuring the validity of the results. The study was conducted in the Department of Anesthesiology, SRM Medical College Hospital and Research Centre, Kattankulathur, Tamil Nadu.

Patients undergoing elective spine surgery under general anesthesia were randomized using computer-generated random numbers into groups "Group A" and "Group B" with 30 patients in each group with odd numbers allotted to Group A and even numbers allotted to Group B. Group A received an ESPB, while Group B received a TLIPB. Under a rigorous standard monitoring protocol, patients were given anesthesia. Patients were premedicated with 0.02 mg/kg glycopyrrolate, 0.1 mg/kg midazolam, and ondansetron 0.1 mg/kg. Patients received fentanyl at 2 mg/kg for analgesia; induction was done with propofol at 2 mg/kg followed by administration of vecuronium at 0.1 mg/kg or atracurium at 0.5 mg/kg as a muscle relaxant. Following this, patients were intubated with appropriate-size endotracheal tubes. Anesthesia throughout the surgery was maintained with 50% oxygen, 50% Nitrous oxide, and 1-2 MAC sevoflurane. Patients were put in the prone position and the block was performed by an anesthesiologist trained in regional anesthesia. ESP block was given in Group A patients using a high-frequency linear ultrasound probe by injecting 40 mL of 0.375% of ropivacaine on each side. Similarly, a TLIP block was performed in Group B aseptically using a high-frequency linear probe at the level of L3 vertebrae. About 40 mL of 0.375% ropivacaine (20 mL on each side) was given in between the longissimus and multifidus muscle and iliocostalis.

All the outcomes were recorded by an anesthesiologist in the postoperative care unit. Intraoperative extra doses of fentanyl, the first postoperative request for analgesia, and the total analgesic consumption during the first postoperative 24 hours were recorded. Intravenous morphine was used postoperatively for pain management and the dose was determined using a visual analog scale (VAS). Data were reported as mean, standard deviation, frequency, and percentage. Continuous variables were analyzed with the independent sample t-test, while categorical variables were assessed using the Pearson Chi-square test. Statistical significance was set at p-values less than 0.05 with a two-tailed test. The data analysis was conducted using IBM-SPSS version 21.0 (IBM Corp., Armonk, NY, USA).

## Results

Sixty patients were randomly assigned into two equal groups, Group A and Group B. Both groups were matched regarding demographic data. The mean age in Group A (Mean±SD: 47.73±10.29) was higher than Group B (Mean±SD: 43.93±9.87). There were 50% male and 50% female in Group A, while 73.3% were female and 26.7% male in Group B. 3.3% of patients were assessed as ASA grade 1 in Group A, while 6.7% were assessed in Group B. Additionally, 96.7% of patients were evaluated as ASA Grade 2 in Group A, while 93.3% were evaluated in Group B (Table 1).

**Table 1:** Baseline characteristics and postoperative outcomes of study participants ASA: American Society of Anesthesiologists

		Group A	Group B	P-value
Age (mean±SD)		47.73±10.29	47.73±10.29	0.15
Sex		Sex (N%)	Sex (N%)	0.063
	Female	15 (50.0)	22 (73.3)	
	Male	15 (50.0)	8 (26.7)	
ASA grade		ASA Grade (N%)	ASA Grade (N%)	0.554
	1	1 (3.3)	2 (6.7)	
	2	29 (96.7)	28 (93.3)	
Postoperative morphine consumption in mg (mean±SD)		8.30±2.89	6.43±2.85	0.015
Duration of analgesia in minutes (mean±SD)		505.00±167.88	435.33±181.01	0.128

The mean postoperative morphine consumption(in mg) was significantly higher in Group A, 8.30±2.89, compared to 6.43±2.85 in Group B. The mean duration of analgesia (in min) was higher in Group A (505±167.88) compared to Group B (435.33±181.01) (Table 1).

**Figure 1:**
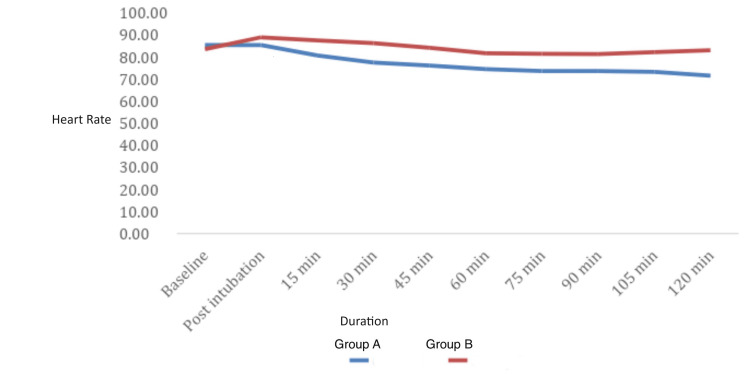
Graph containing mean heart rate in Group A and Group B

The heart rate was recorded at baseline, post-intubation, after 15 min, 30 min, 45 min, 60 min, 75 min, 90 min, 105 min, and 120 min in both groups. The mean heart rate has been calculated and data has been shown in Figure 1.

**Figure 2:**
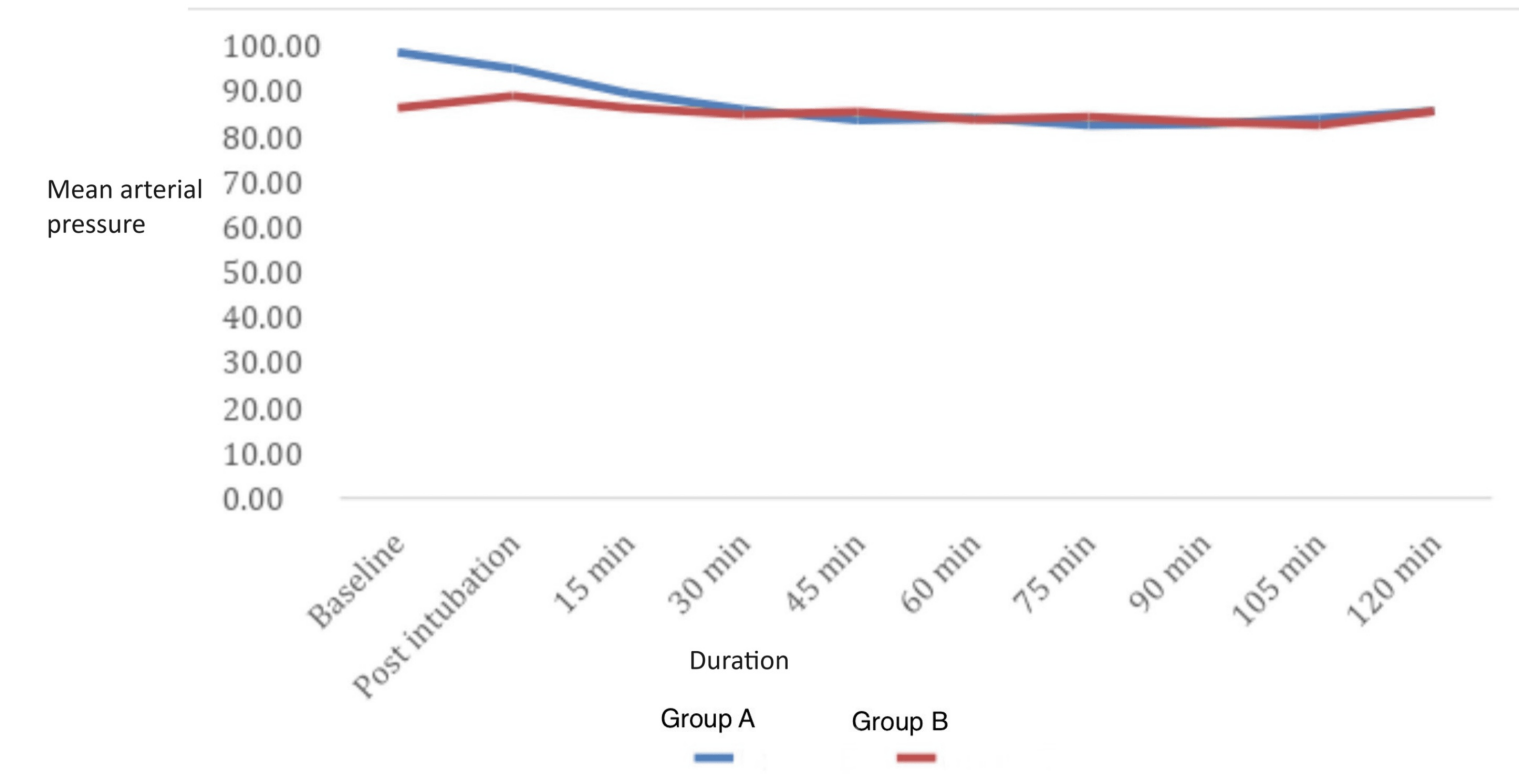
Graph comparing mean arterial pressure in Group A and Group B

The mean arterial pressure was recorded at baseline, post-intubation, at 15 min, 30 min, 45 min, 60 min, 75 min, 90 min, 105 min, and 120 min in both groups. The mean arterial pressure has been calculated and data are shown in Figure 2.

**Figure 3:**
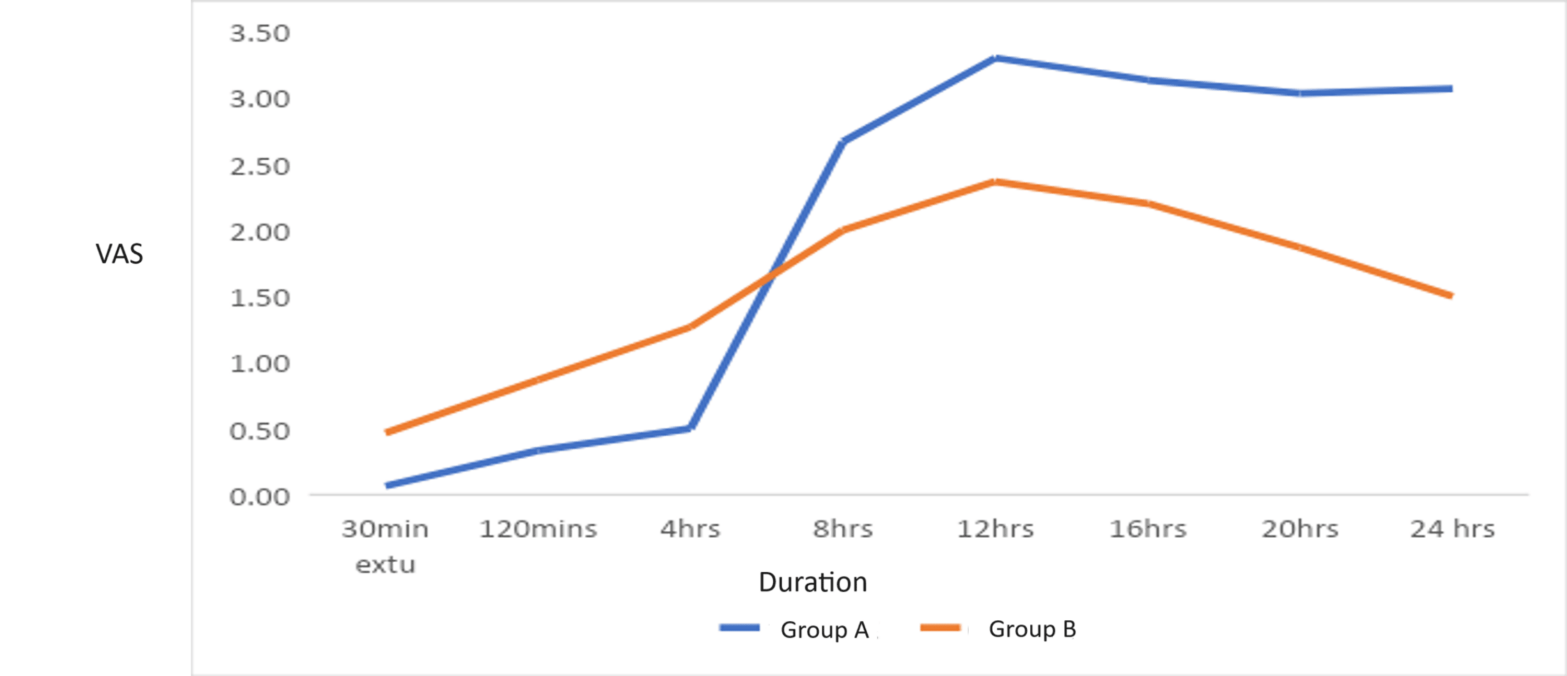
Graph comparing VAS in Group A and Group B VAS: visual analog scale

The VAS has been used to assess the level of pain in both groups post-extubation at the interval of 30 min, 120 min, 4 hours, 8 hours, 12 hours, 16 hours, 20 hours, and 24 hours. The mean value has been calculated and is shown in Figure 3.

## Discussion

This study presents a randomized control trial comparing the efficacy of ultrasound-guided ESPB with TLIPB for lumbar spine surgeries. Both blocks are increasingly utilized as part of multimodal analgesia strategies in various surgeries. The trial involved assessing pain scores, opioid consumption, and perioperative outcomes in patients undergoing lumbar spine surgeries. Both techniques exhibited satisfactory safety profiles. This study provides valuable insights into the optimal regional anesthesia technique for improving postoperative pain management in lumbar spine surgeries, contributing to enhanced patient satisfaction and faster recovery.

Both the ESPB and TLIPB provided significant analgesia. Still, the mean duration of analgesia is greater with EPSB compared to TLIPB, which is consistent with the study done by Pelin Dilsiz et al., where 68 patients who took elective lumbar disk surgery were randomly assigned to either the ESPB group or the TLIPB group [6]. The patient's pain status in the ESPB and TLIPB groups was evaluated utilizing the Numerical Rating Scale (NRS) at fixed time intervals (30 min, 1 hour, 6 hours, 12 hours, and 24 hours) during the postoperative period. In their study in the ESPB group, the total opioid consumption as morphine equivalents was found to be lower (ESPB group: 7.7±7.0; TLIPB group: 13.0±10.1; p<0.05). The NRS scores were comparable between the groups at 30 min, 1 hour, 6 hours, and 12 hours, but at 24 hours, they were lower in the ESPB group. Moreover, the groups had no significant difference regarding observed side effects. The number of times patients were given a bolus dose of patient-controlled analgesia (PCA) in the first 24 hours was noted.

In ESPB, it is understood that the local anesthetic affects both the dorsal and ventral branches of the spinal nerves, resulting in a sensory block that spans multiple dermatomes, which is consistent with the study done by HM Yang et al. [7]. In their cadaveric study, they compared the anatomical spread of dye in the thoracic region by following these two procedures.

The mean pain scores and opioid consumption were lower in the TLIPB, which was also seen in the study done by Ahmet Kaciroglu et al. [8]. However, in their study, they compared the ESPB and TLIPB with the control group, and it was found that the opioid consumption and mean pain scores were lower in the TLIPB group. The TLIP block could offer more targeted analgesia compared to the ESPB for lumbar spine surgeries. Since the TLIP block specifically targets the dorsal rami, it may be an optimal analgesic approach for procedures involving the lumbar region, which was also concluded in a study done by Bahadir Ciftci et al. [9].

The limitation of the study was the use of a linear probe instead of a curvilinear probe. Had it been a curvilinear probe it would have led to better visualization of the structures. Another limitation of the study was TLIPB, which requires expertise and is a more complex technique when performed. This study was based on a single center. Hence, we require a multicentric trial involving different population groups to have a better understanding.

## Conclusions

Based on visual analog pain score and postoperative opioid consumption, TLIPB emerges as a superior option over ESPB for managing postoperative pain in significant spine surgeries. While ESPB provides dermatomal coverage for both the dorsal and ventral rami of spinal nerves, the local anesthetic spreads in both anterior and posterior directions, potentially leading to delayed pain sensation. In contrast, TLIPB is administered between the longissimus and iliocostalis muscles, primarily targeting the dorsal rami of spinal nerves and offering denser dermatomal coverage crucial for spine surgeries. Consequently, patients receiving TLIPB may experience earlier pain sensations, but they exhibit lower VAS scores and require less postoperative opioid medication compared to those receiving ESPB. Ultimately, the choice between these techniques remains with the anesthesiologist, who will decide based on their experience and ease of putting block.
